# Breast PET/MRI Hybrid Imaging and Targeted Tracers

**DOI:** 10.1002/jmri.28431

**Published:** 2022-09-27

**Authors:** Valeria Romeo, Thomas H. Helbich, Katja Pinker

**Affiliations:** ^1^ Department of Advanced Biomedical Sciences University of Naples Federico II Naples Italy; ^2^ Division of General and Pediatric Radiology, Department of Biomedical Imaging and Image‐Guided Therapy Medical University of Vienna Wien Austria; ^3^ Department of Radiology, Breast Imaging Service Memorial Sloan Kettering Cancer Center New York New York USA

**Keywords:** breast cancer, magnetic resonance imaging, positron emission tomography

## Abstract

The recent introduction of hybrid positron emission tomography/magnetic resonance imaging (PET/MRI) as a promising imaging modality for breast cancer assessment has prompted fervent research activity on its clinical applications. The current knowledge regarding the possible clinical applications of hybrid PET/MRI is constantly evolving, thanks to the development and clinical availability of hybrid scanners, the development of new PET tracers and the rise of artificial intelligence (AI) techniques. In this state‐of‐the‐art review on the use of hybrid breast PET/MRI, the most promising advanced MRI techniques (diffusion‐weighted imaging, dynamic contrast‐enhanced MRI, magnetic resonance spectroscopy, and chemical exchange saturation transfer) are discussed. Current and experimental PET tracers (^18^F‐FDG, ^18^F‐NaF, choline, ^18^F‐FES, ^18^F‐FES, ^89^Zr‐trastuzumab, choline derivatives, ^18^F‐FLT, and ^68^Ga‐FAPI‐46) are described in order to provide an overview on their molecular mechanisms of action and corresponding clinical applications. New perspectives represented by the use of radiomics and AI techniques are discussed. Furthermore, the current strengths and limitations of hybrid PET/MRI in the real world are highlighted.

**Evidence Level:**

2

**Technical Efficacy:**

Stage 2

Hybrid PET/MRI represents the new frontier in cancer imaging. In recent years, it has been established that cancer is a highly heterogeneous disease, and despite well‐established cancer molecular patterns and biology, each patient remains unique in terms of disease behavior and prognosis. In treating patients with solid tumors, information regarding their tumors mainly comes from imaging of the tumor throughout the treatment continuum; thus, imaging modalities that provide not only morphological, but also functional data, are particularly valuable.^1^, ^2^

Breast cancer is the most common solid tumor among women. Breast cancer exemplifies cancer heterogeneity as it is characterized by different molecular patterns which are associated with different treatment options and prognoses.^3^ Among the available imaging modalities to image breast cancer, MRI is the most sensitive.^4^ In addition to allowing the simultaneous evaluation of both breasts, MRI allows for a comprehensive morphological and functional assessment, including the assessment of tumor neoangiogenesis (via a dynamic contrast‐enhanced [DCE] sequence) and cellularity (via a diffusion‐weighted imaging [DWI] sequence). Through the application of pharmacokinetic models, perfusion parameters can also be extracted and quantified as measures of tumor permeability reflecting the exchanges of the contrast agent between blood vessels and the surrounding interstitium.^5^ MRI parameters have proven to be effective in depicting tumor aggressiveness as well as in assessing and monitoring response to systemic treatment.^6^, ^7^

In view of the eminent role of MRI in breast cancer assessment, the combination of MRI with PET as a hybrid imaging tool would seem especially promising, opening up new research avenues for improving patient care and management even further.^8^, ^9^ Recent and ongoing investigations concerning functional imaging have sought to noninvasively identify and monitor cancer processes at the molecular level.^10^ While 2‐(18F)fluoro‐2‐deoxy‐d‐glucose (^18^F‐FDG) PET has an established role in clinical practice, investigations to identify novel PET radiotracers for visualizing new molecular targets are underway, which may lead to improvements in breast cancer characterization, treatment stratification, and response prediction and assessment. In light of the availability of different PET tracers targeting different biological tumor properties, hybrid PET/MRI seems poised to become the best imaging technique to comprehensively describe molecular processes underlying cancer development—i.e., those “hallmarks of cancer” (Fig. 1).

**FIGURE 1:**
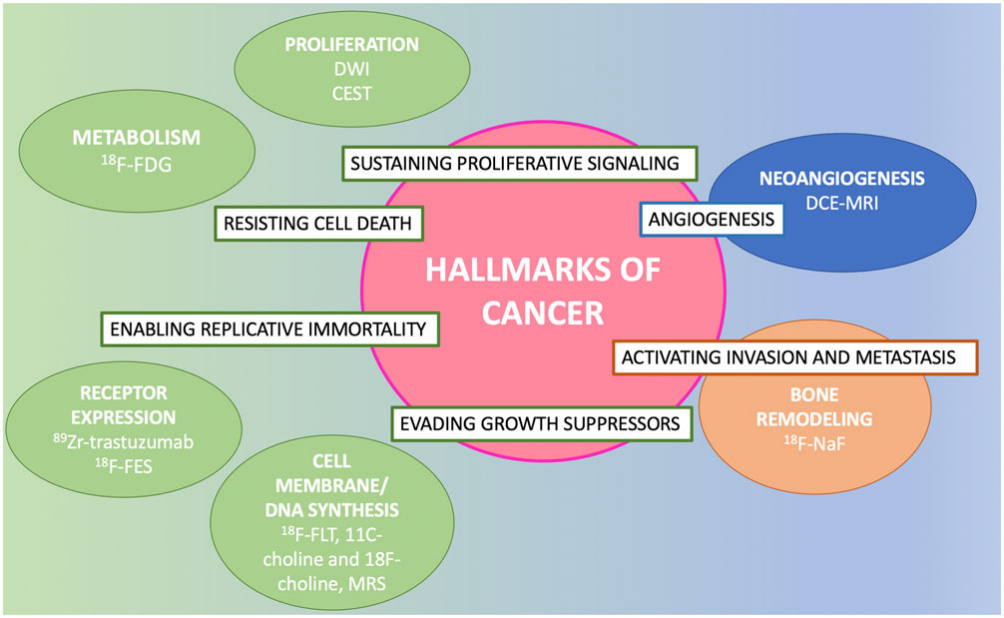
Hallmarks of cancer with corresponding PET and MRI biomarkers.

The aim of this review is to present up‐to‐date evidence on the role of hybrid PET/MRI in breast cancer assessment. The first section provides an overview of the technical aspects of hybrid PET/MRI. New advances in both MRI and PET allowing for molecular‐level assessment of breast tissue will also be described. The second section is dedicated to describing novel PET tracers, including their mechanisms of action and corresponding biological implications and clinical applications. The third section provides evidence for clinical applications of hybrid PET/MRI of the breast. Take‐home points and illustrations are provided to better summarize and illustrate the main concepts.

## Technical Aspects

### Where the Story Begins

In 2010, the first hybrid PET/MRI scanners were installed for clinical use at Mount Sinai Hospital in New York, NY, USA, and at Technical University of Munich, in Germany. Several technical challenges had to be overcome in order to combine the two modalities. On one hand, PET detectors caused interferences in MR magnetic field homogeneity, radiofrequency, and gradient systems.^11^ On the other hand, eddy currents, which are undesired currents generated by changes in the magnetic field and radiofrequency pulse, affect PET signal detection. To combine the two modalities effectively, MRI‐ and PET‐compatible devices such as avalanche photodiode‐based PET detectors (i.e., highly sensitive detectors with internal gain produced by the application of a reverse voltage) were developed. Furthermore, strategies for reducing the effect of eddy currents to PET analysis were put into place such as covering PET detector modules with copper foil. An alternative was to collect PET data sequentially by disabling the PET detectors during MRI acquisition.^12^ Of note, to combine the two modalities effectively, attenuation correction of PET images is also needed, but MR images, which reflect information on proton density, do not provide linear attenuation coefficients. An alternative to attenuation correction is to use Dixon sequences which generate fat and water images and allow the segmentation of four body compartments such as fat, soft tissue, lung, and air with corresponding linear attenuation coefficients.^13^

Hybrid PET/MRI of the breast allows for the simultaneous collection of morphologic, functional, and metabolic information, not only of the breast but also of the whole body in a single examination, thus providing relevant diagnostic, prognostic, and predictive information. While PET can be combined with either CT (hybrid PET/CT) or MRI (hybrid PET/MRI) with precise time matching, MRI provides superior soft tissue visualization and additional functional imaging capability compared to CT. The PET/MRI examination for breast cancer evaluation is typically made of two distinct examinations: contrast‐enhanced dedicated breast PET/MRI, acquired with patient in the prone position, followed by the whole‐body acquisition, with the patient lying in the supine position. Both examinations are described below.

### Breast PET/MRI Acquisition Protocol: The Basics

Patients are first injected with the selected radiotracer. If the ^18^F‐FDG radiotracer is used, patients will need to fast for 5–6 hours prior to breast PET/MRI. Then, according to the timing of the tracer's distribution within the body, the patient is positioned in the prone position in the MRI gantry, with both breasts positioned in the dedicated breast coil. Breast PET/MRI should begin with the acquisition of conventional MRI sequences, such as T2‐weighted imaging with and/or without fat suppression, DWI, and volumetric DCE imaging. All sequences are usually acquired in the axial plane. A late T1‐weighted sequence with fat suppression on coronal plane is also recommended for the assessment of axillary regions.

**FIGURE 2:**
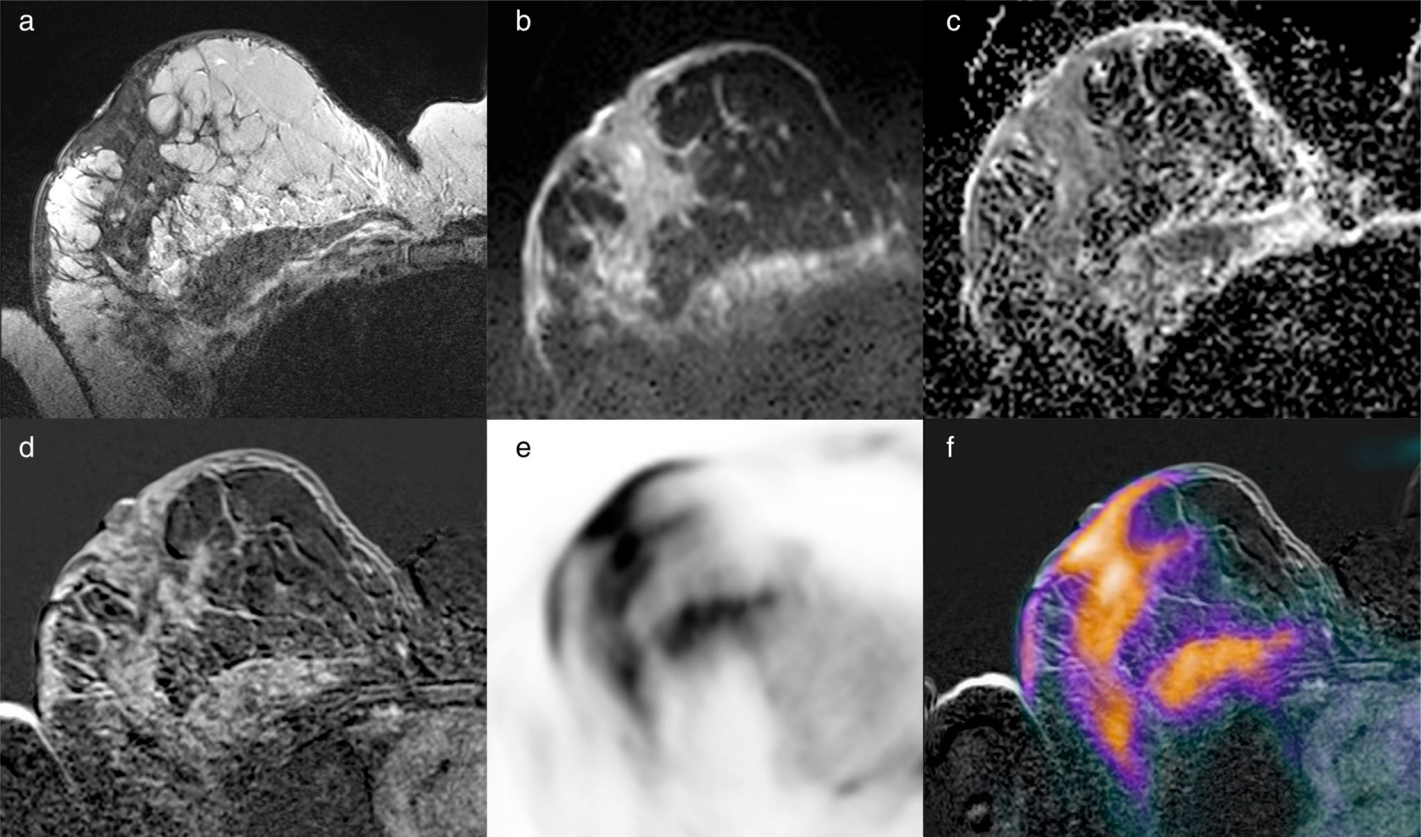
An example of multiparametric imaging obtained using hybrid ^18^F‐FDG PET/MRI. A 56‐year‐old woman with invasive ductal breast cancer (G3, triple negative) in the right breast associated with pectoral muscle infiltration and skin thickening, shown on axial **(a)** fat‐saturated T2‐weighted turbo spin‐echo imaging, **(b)** diffusion‐weighted echo‐planar imaging and **(c)** apparent diffusion coefficient mapping, **(d)** contrast‐enhanced T1‐weighted imaging, **(e)** PET imaging, and **(f)** fused PET/MRI imaging.

DWI can be performed either before or after contrast agent administration, as its timing does not affect apparent diffusion coefficient (ADC) calculations. However, it is worth noting that post‐contrast ADC parameters can be slightly lower than pre‐contrast ADC parameters, due to susceptibility artifacts, so that pre‐contrast acquisition is preferred.^14^, ^15^ DCE‐MRI, performed before and after paramagnetic contrast agent administration, can be acquired according to the routine protocol, consisting of four to five post‐contrast acquisitions, with a total acquisition time of ~20 minutes or, alternatively and usually in a research setting, using ultrafast perfusion imaging, as outlined further below. The entire protocol is therefore multiparametric in nature and includes the acquisition of hybrid images derived from the fusion of MRI and PET images (Fig. 2).

### Whole‐Body Imaging

**FIGURE 3:**
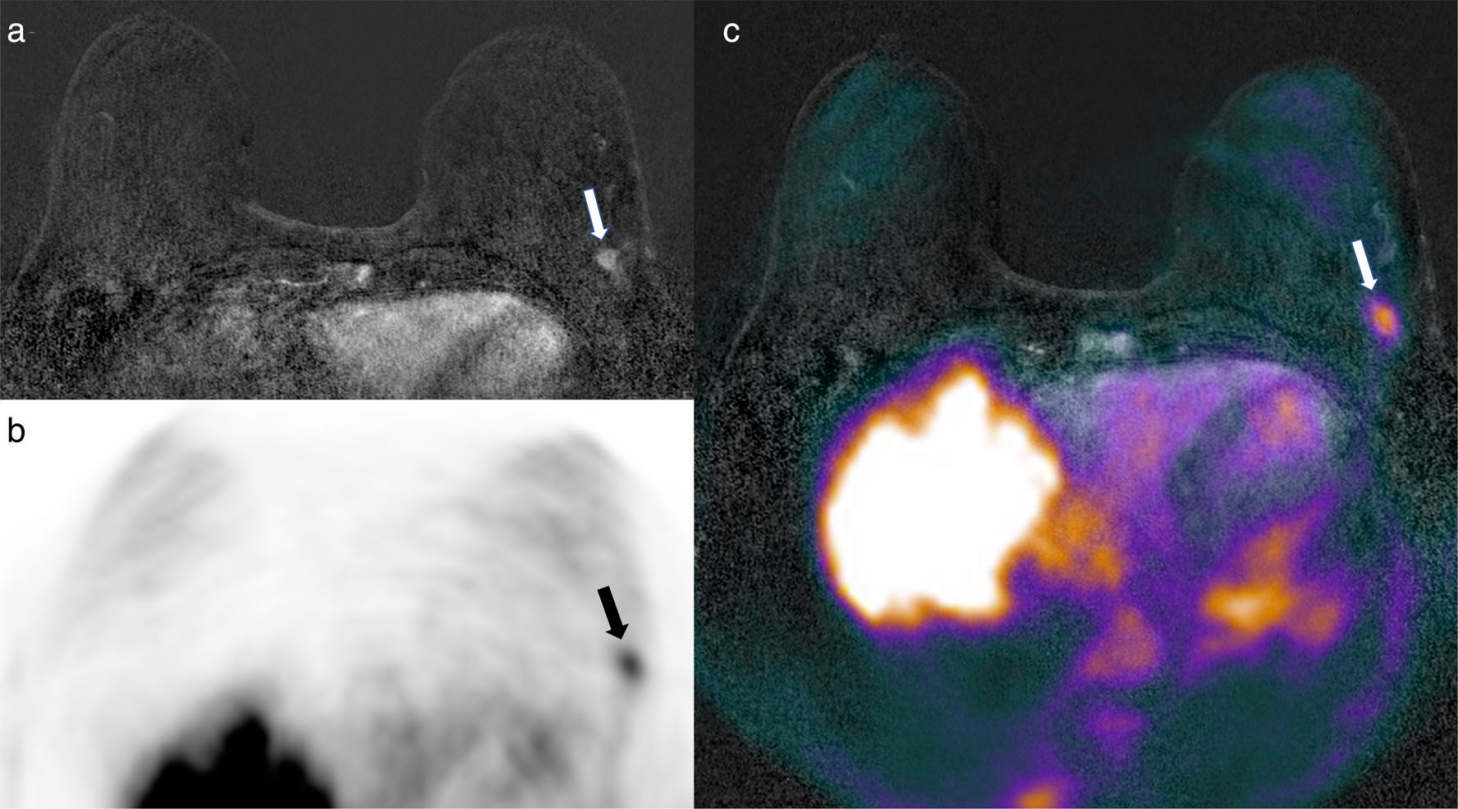
Axial **(a)** subtracted dynamic contrast‐enhanced (DCE)‐MRI, **(b)** PET, and **(c)** fused DCE‐MRI and PET imaging. A 66‐year‐old patient with invasive ductal breast cancer (9 mm, G3, ER/PgR−, HER2+) of the left breast (arrows in **a**–**c**).

**FIGURE 4:**
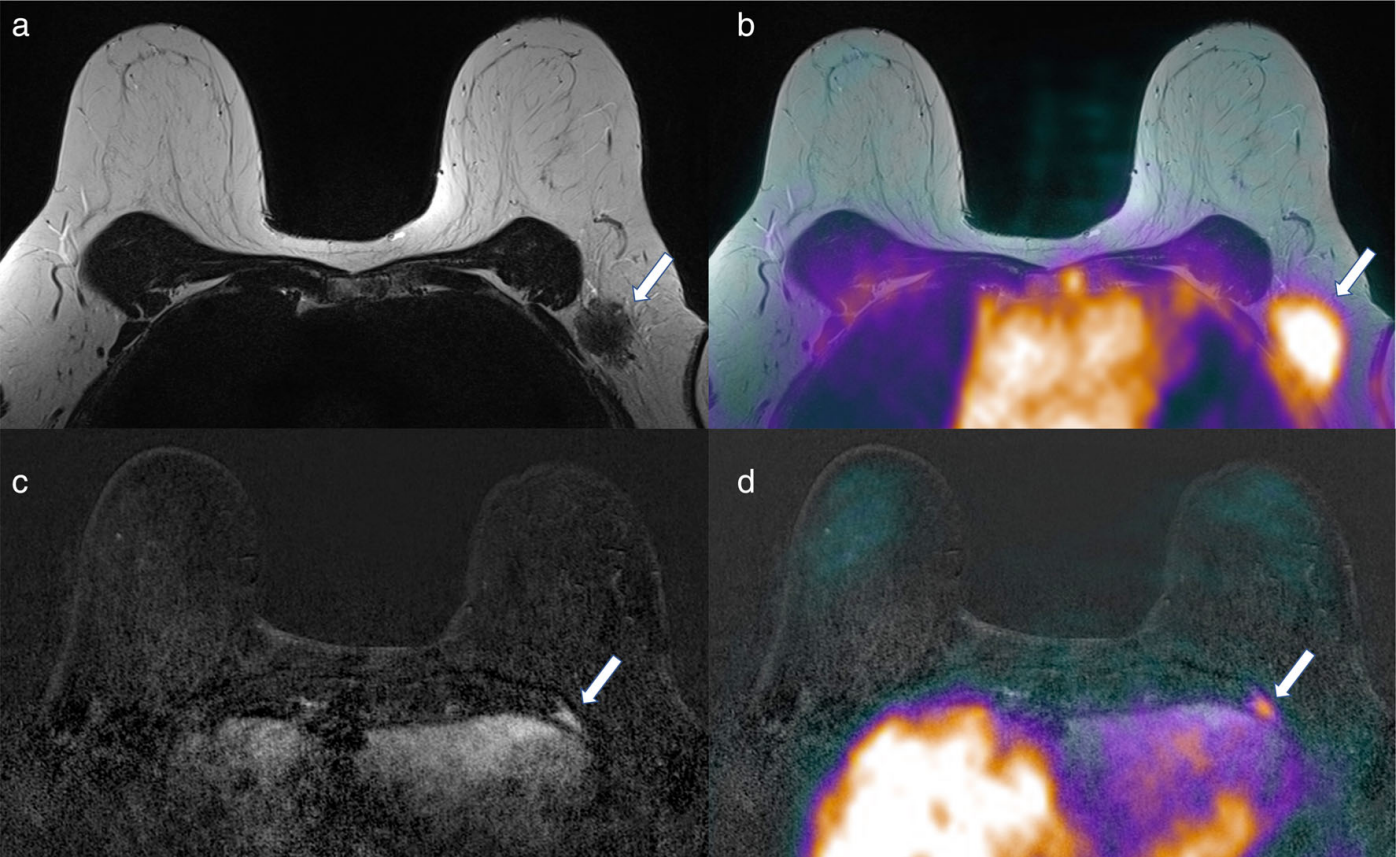
Axial **(a)** T2‐weighted, **(b)** fused T2‐weighted and PET, **(c)** subtracted DCE‐MRI, and **(d)** fused DCE‐MRI and PET imaging. A heterogeneous axillary metastasis (arrows in **a** and **b**) and a rib bone metastasis (arrows in **c** and **d**) are detectable in a 66‐year‐old patient with invasive ductal breast cancer (G3, ER/PgR−, HER2+) of the left breast (same patient as Fig. 3).

**FIGURE 5:**
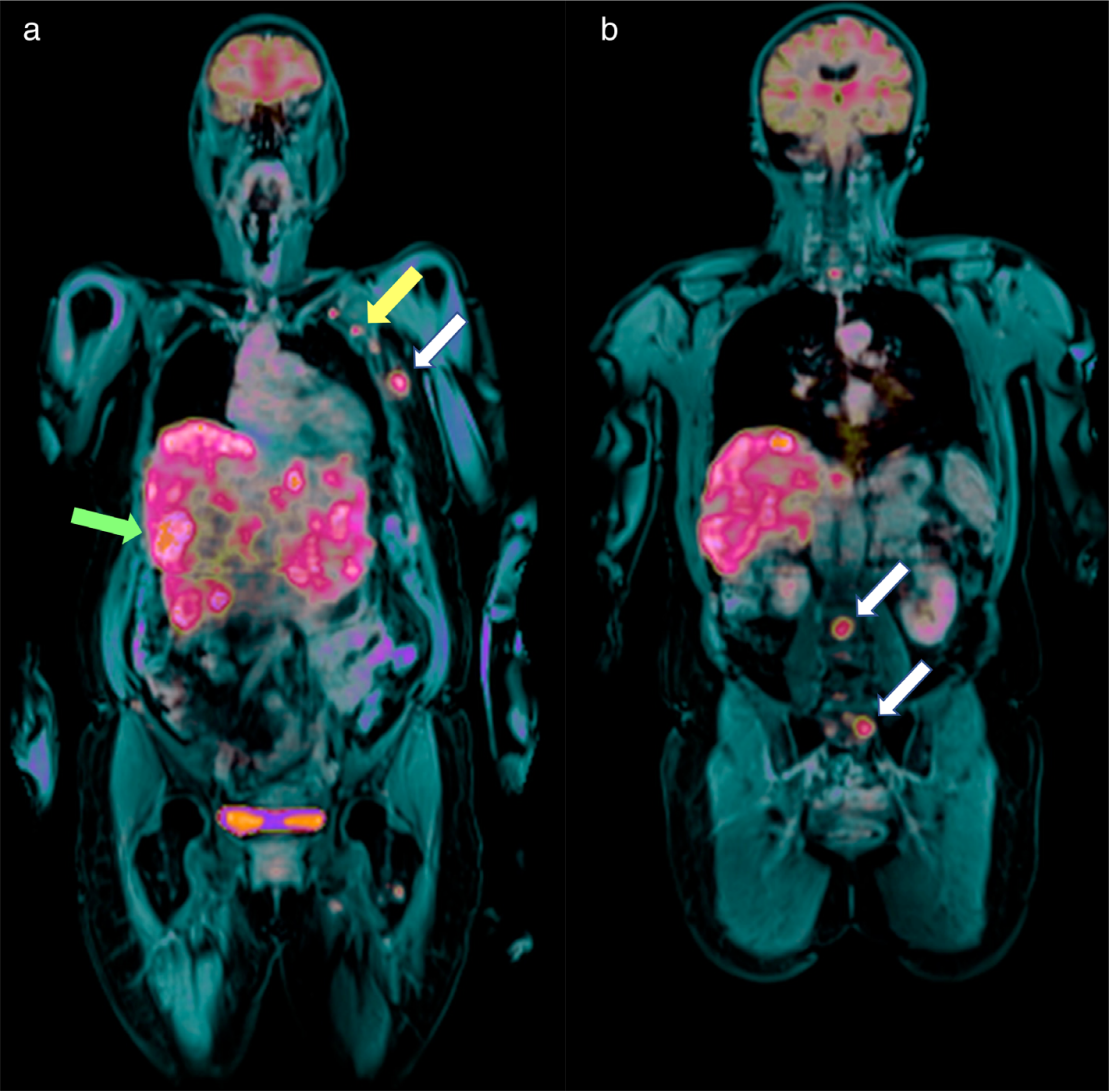
(**a** and **b**) Fused PET and post‐contrast fat‐saturated T1‐weighted imaging on the coronal plane (whole‐body examination) shows liver and axillary involvement (green and yellow arrows in **a**, respectively) as well as rib and lumbo‐sacral bone metastases (white arrows in **a** and **b**, respectively) in in a 66‐year‐old patient with invasive ductal breast cancer (G3, ER/PgR−, HER2+) in the left breast (same patient as the patient in Figs. 3 and 4).

**FIGURE 6:**
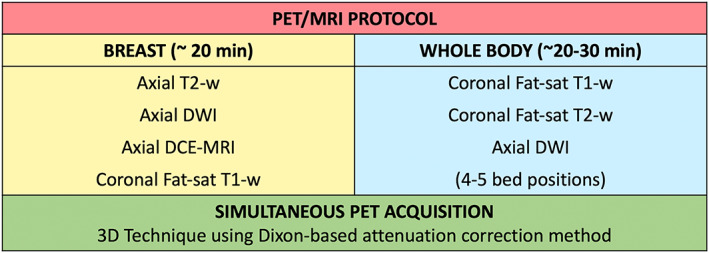
Illustration of breast and whole‐body hybrid PET/MRI acquisition protocols.

Besides the dedicated breast protocol, the PET/MRI protocol for breast cancer assessment can also include whole‐body imaging, especially when performed for staging and treatment assessment purposes. The coronal plane is preferred, especially for T2‐weighted imaging, usually with fat suppression, and for gradient‐echo, fat‐suppressed T1‐weighted imaging, exploiting the contrast given by the gadolinium injection performed during the prior breast examination. DWI acquisition is usually performed in the axial plane. The patient lies in the supine position, with head and body coils positioned, and four to five “bed positions” are acquired along with PET data. Similar to MRI acquisition, no further ^18^F‐FDG is injected in addition to that employed for the breast protocol. The final whole‐body image is obtained by combining all segments. The whole‐body protocol should be as fast as possible, considering issues related to claustrophobia and the positioning of all coils on the patient's body simultaneously. Several efforts have been made in this direction, with the total acquisition time being kept around 20 minutes for assessing pelvic malignancies.^16^, ^17^ An example of a breast and whole‐body hybrid 18F‐FDG PET/MRI examination in a patient with a small but aggressive invasive ductal carcinoma (G3, ER/PgR/HER2−) presenting with axillary and distant metastases in given in Figs. 3, 4, 5, while a summary of breast and whole‐body PET/MRI protocols is illustrated in Fig. 6.

### Advanced MRI Tools: Toward Molecular‐Level Characterization

#### Ultrafast DCE‐MRI

Ultrafast DCE‐MRI with a high temporal resolution (preferably <10 sec) may be performed, preceded by T1 mapping for tissue T1 quantification.^18^ In the research setting, this approach has been shown to allow for the calculation of perfusion quantitative parameters via a pharmacokinetic model, usually Tofts,^5^ reflecting contrast agent exchanges between tumor vessels (plasma) and neighboring extravascular, extracellular space (EES), as a measure of tumor permeability. Among the most widely used perfusion parameters, K^trans^ describes the efflux of contrast from the plasma to the EES, while K_ep_ is a measure of contrast influx from the EES to the plasma; v_e_ expresses the volume of the EES, which can be considered as a measure of cell density, and v_p_ represents the plasma volume.^19^ Recommendations for both perfusion technique and parameter calculation have been published,^20^, ^21^ albeit further efforts are necessary to standardize parameter calculation.^22^

#### Advanced DWI Applications

Preliminary data are currently available on the usefulness of intravoxel incoherent motion (IVIM) and diffusion tensor imaging (DTI) in breast imaging.^23^ IVIM is a noninvasive method for the discrimination of blood microcirculation (pseudo‐diffusion) and true molecular diffusion, providing perfusion and diffusion‐related quantitative parameters (D or Dt and D*, Dp or Df, respectively). Of note, some of these perfusion parameters were found to be more accurate than ADC for discriminating benign from malignant breast lesions^24^ and were also correlated with breast cancer prognostic factors.^25^, ^26^

DTI is a noninvasive method which assesses the directional diffusivity of water molecule in biological tissues. DTI has been applied to breast imaging based on the concept that breast cancer destroys the ductal organization, thus reducing its anisotropy.^23^ Further studies are, however, necessary to prove this hypothesis and determine the applicability of DTI to breast cancer assessment.

#### ^1^H MR Spectroscopy

**FIGURE 7:**
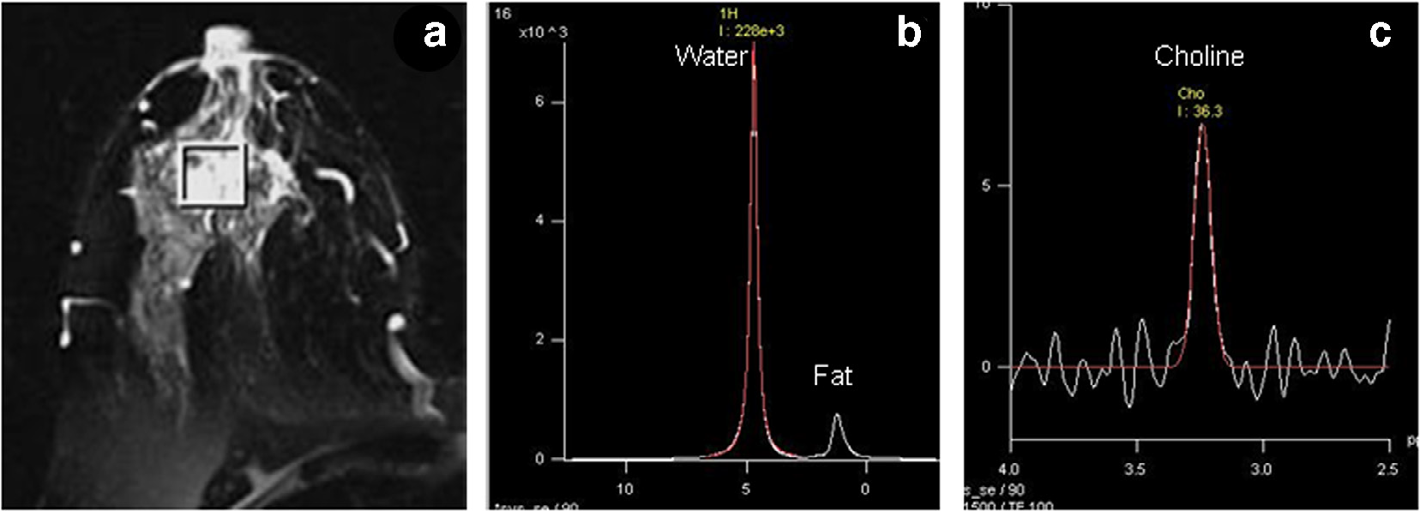
**(a)** T2 weighted MR image of a patient suffering from locally advanced breast cancer while **(b)** shows the in vivo 1H MR spectrum acquired without water and fat suppression from the VOI shown in **(a)**. **(c)** MR spectrum obtained from the same voxel with water + fat suppression. VOI, volume of interest. Reprinted under a Creative Commons (CC BY 4.0) license from: Sharma U, Jagannathan NR. In vivo MR spectroscopy for breast cancer diagnosis. BJR Open. 2019;1(1):20180040. doi: 10.1259/bjro.20180040. PMID: 33178927; PMCID: PMC7592438.

Breast PET/MRI can also be enriched with further advanced and novel sequences, including proton MR spectroscopy (^1^H MRS) and chemical exchange saturation transfer (CEST) imaging.^27^, ^28^, ^29^ ^1^H MRS is based on the concept that protons excited by a radiofrequency pulse resonate at different frequencies depending on their chemical environment, thus allowing the concentration of different metabolites within a region of interest to be determined.^30^ In ^1^H MRS, after the perturbation of the magnetic field due to an RF pulse, an MRI frequency spectrum is obtained where chemical compounds are represented based on their specific frequency, expressed in parts per millions (ppm) (Fig. 7). To date, the choline metabolite has been shown to be overrepresented in breast cancer due to high membrane turnover, so that its peak (encountered at 3.2 ppm) may be identified and quantified for different clinical purposes (eg, breast cancer diagnosis or chemotherapy monitoring).^31^, ^32^

#### Chemical Exchange Saturation Transfer (CEST)

**FIGURE 8:**
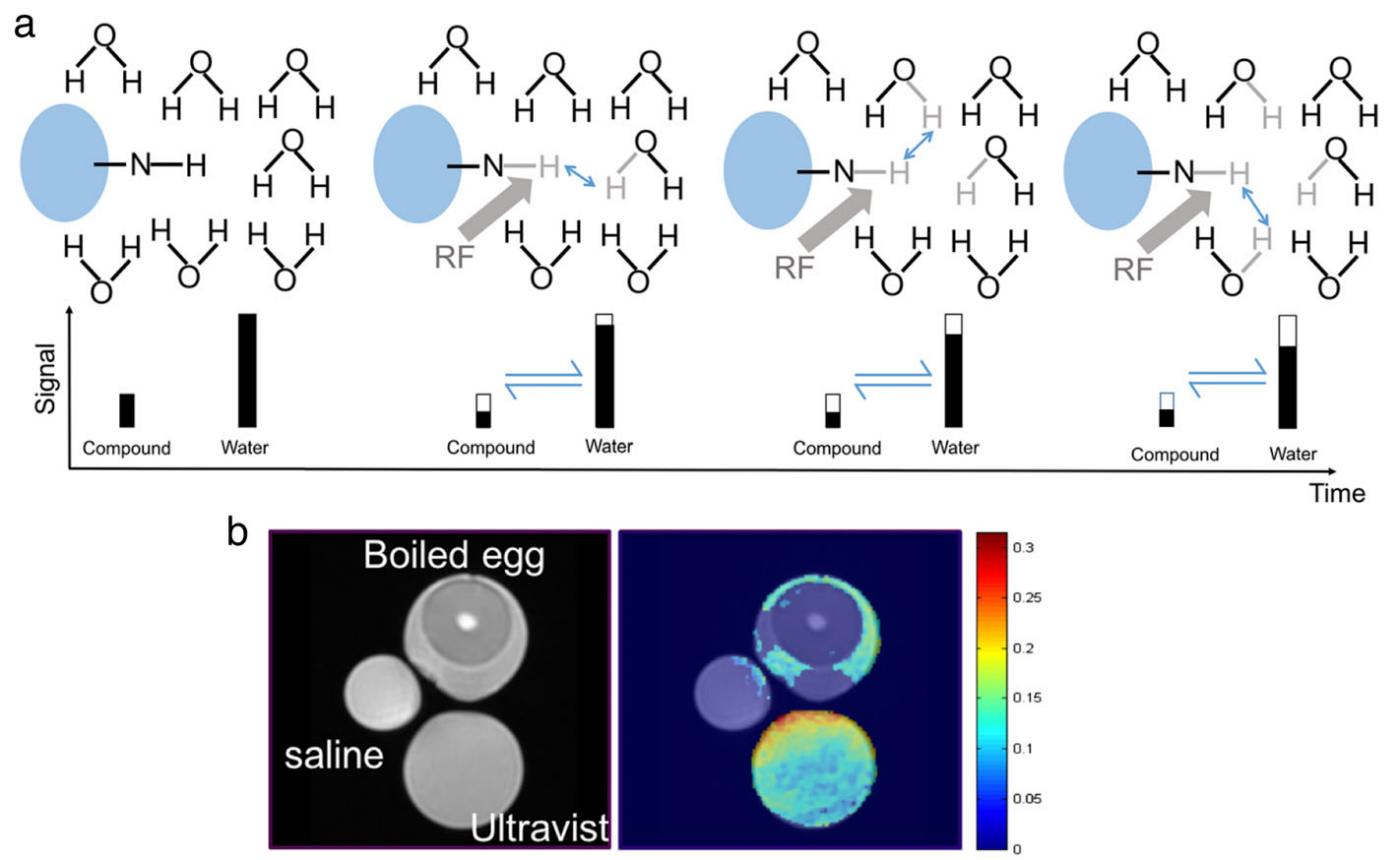
**(a)** Diagram illustrating the process of chemical exchange saturation transfer (CEST): in a solute, the small quantity of chemical substance containing an amine group (‐NH) is saturated by a RF, which initially reduces the signal of the substance (shown as the hollow bar); then, the saturated hydrogen proton is transferred to water in return for an unsaturated hydrogen; this process continues that leads to amplified water signal reduction (assumes that the saturation level on the chemical substance itself remains unchanged). This process will continue subject to the T1 relaxation and back exchange. **(b)** Comparison between conventional T2‐weighted image and CEST at 4.2 ppm: only Ultravist (Iopromide solution) and egg white yielded CEST contrast. Reprinted under a Creative Commons (CC BY 4.0) license from: Wu B, Warnock G, Zaiss M, Lin C, Chen M, Zhou Z, Mu L, Nanz D, Tuura R, Delso G. An overview of CEST MRI for non‐MR physicists. EJNMMI Phys. 2016;3(1):19.

In CEST imaging, endogenous compounds containing exchangeable protons which are too small in concentration for their detection by either conventional MRI or MRS are selectively detected. The saturation of these protons, obtained through a radiofrequency pulse applied at their resonance frequency, is spontaneously transferred to the surrounding water, allowing for their indirect visualization and concentration estimation.^28^ An illustration of the CEST process is provided in Fig. 8. Amide CEST, also known as amide proton transfer, allows for the identification of proteins, peptides, and amino acids, which are usually present at high concentrations in tumor regions. Considering the possible inclusion of these additional sequences, the total acquisition time of the breast examination becomes highly variable.

## PET Tracers

PET tracers consist of a positron‐emitting isotope bound to an organic ligand which is able to interact with a target protein (eg, glucose transporter, hormone receptor). Once injected in the bloodstream, the distribution of the tracer reflects that of the target protein, revealing where in the body its specific biological process is occurring. In this section, PET tracers are discussed, from routinely used tracers to the most promising experimental ones. For each tracer, the mechanism of action with corresponding biological implications and clinical applications are highlighted. A summary of these data can be found in Table 1.

**TABLE 1:** Summary of PET tracers most employed/investigated for breast imaging, with corresponding biological properties and clinical applications

Tracer	Full Name	Detected Biological Processes	Clinical Use	FDA Approval
^18^F‐FDG	2‐deoxy‐2‐18Ffluoroglucose	GLUT‐1 upregulation Hexokinase activity	Staging Response assessment Diagnosis	Yes
^18^F‐NaF	Fluorine 18–Sodium Fluoride	Hydroxyapatite exposure during bone remodeling	Detection of bone metastasis	Yes
^18^F‐FES	16α‐18F‐fluoroestradiol	Estrogen receptor expression	Staging Diagnosis ER+ BC	Yes
^89^Zr‐trastuzumab	^89^Zr‐trastuzumab	HER2 expression	Diagnosis HER+ BC Assessment HER2 status	No
^11^C‐choline and 18F‐choline	N‐[11C] methylcholine 18F‐Fluoroethylcholine	Cell membrane synthesis	Staging Response assessment Diagnosis	Yes
^18^F‐FLT	3‐deoxy‐3‐[18F] Fluorothymidine	DNA synthesis	Response assessment	No
^68^Ga‐FAPI‐46	^68^Ga‐conjugated fibroblast activation protein inhibitor	FAP detection‐ modulation of tumor microenvironment	Diagnosis Staging	No

### Which Tracers Can Be Employed in Clinical Practice?

At present, PET tracers employed for breast cancer and approved for clinical use are: ^18^F‐FDG, sodium fluoride labeled with fluorine (^18^F‐NaF), Carbon‐11 choline, and Fluorine‐18 fluoroestradiol.

#### ^18^F‐FDG

How it works: ^18^F‐FDG leverages the increased glucose consumption of cancer cells, also known as the “Warburg effect.” GLUT‐1 upregulation causes glucose molecules to be introduced and then trapped inside cancer cells after these molecules are phosphorylated by the hexokinase enzyme. Glucose molecules cannot proceed to glycolysis due to fluorine steric hindrance but, after time decay, they result in glucose 6 phosphate, which can be further metabolized.^33^

When it can be used: ^18^F‐FDG is the most widely used tracer in oncology, with a large number of clinical applications in both solid and hematologic malignancies.^34^ In breast cancer, it is the tracer of choice, with clinical practice guidelines recommending the use of ^18^F‐FDG for tumor staging and restaging of breast cancer (eg, locally advanced breast cancer, particularly aggressive breast cancer subtypes) when distant metastases are suspected corresponding to stage IIIa or greater, or for the assessment of response to systemic treatment.^35^ As most of the evidence presented in the literature for breast PET involves the use of ^18^F‐FDG, the full panel of clinical indications for ^18^F‐FDG PET is extensively described further below along with those of ^18^F‐FDG PET/MRI.

#### ^18^F‐NaF

How it works: During bone remodeling due to either osteolytic or osteoblastic processes, hydroxyapatite is exposed and made available for ion exchange. ^18^F‐NaF leverages this process through the incorporation of ^18^F ions within the bone matrix, a process that also depends on blood flow.^36^, ^37^

When it can be used: ^18^F‐NaF was approved by the US Food and Drug Administration in 1972 following excellent experiences with this tracer for bone metastasis detection. However, it has been gradually supplanted in clinical practice due to the increased availability of gamma cameras and good performance of ^99m^Tc‐MDP. The role of ^18^F‐NaF has been recently re‐discussed in light of the increased availability of PET/CT scanners, in particular if and how ^18^F‐NaF can co‐exist with ^18^F‐FDG. ^18^F‐FDG allows for a comprehensive assessment of both bone and soft tissue, while ^18^NaF is limited to bone evaluation. While further investigations are needed to give a final answer to this question, the current evidence suggests that ^18^F‐FDG is more sensitive for the detection of pure marrow metastasis, while ^18^F‐NaF can be more useful for the detection of malignant processes related to tumors with low FDG uptake, such as renal cancer. Thus, the role of these tracers can be considered complementary, whereby ^18^FDG is used in the initial assessment and ^18^F‐NaF is used to address equivocal issues related to bone involvement.^36^

#### CHOLINE

How it works: Choline represents a marker of cell membrane turnover and thus of cell proliferation. Choline can be labeled with either ^11^C or ^18^F. Of note, ^18^F‐choline has already been used in prostate cancer imaging.^38^

When it can be used: Choline PET imaging shares the same clinical indications as ^18^F‐FDG and thus robust evidence must be produced to assess its additional value to ^18^F‐FDG. Early clinical experiences of ^11^C‐choline show good uptake of ^11^C‐choline in breast cancer cells, with breast cancer cells showing high contrast compared to the surrounding background parenchyma.^39^

In regards to ^18^F‐choline, one of the first evidence of its applicability in breast cancer assessment was the incidental invasive breast cancer finding in a male patient which showed high ^18^F‐choline uptake during a prostate examination.^40^ In a recent study by Wu et al comparing MRS and ^18^F‐choline uptake in 39 benign and malignant breast lesions, a moderate comparison was found between MRS and PET parameters.^41^ Furthermore, in their study, the PET‐based standard uptake value (SUV) obtained with the patient in the supine position was shown to be the best performing parameter for breast cancer diagnosis, with an area under the curve (AUC), sensitivity, and specificity were 0.918, 89.5%, and 87.5%, respectively, using a cut‐off of 2.5. Clinical investigations for the feasibility and usefulness of ^18^F‐choline PET/MRI of the breast are currently ongoing.

#### ^18^F‐FES

How it works: ^18^F‐FES is a derivative of estrogen and is meant to bind to estrogen receptors which are overexpressed in luminal breast cancers.^42^, ^43^

When it can be used: Estrogen‐positive breast cancer is the most common breast cancer molecular subtype, accounting for 50%–60% of breast malignancies.^44^ Reasons for the use of ^18^F‐FES range from diagnosis to local and distant staging as well as assessing response to treatment in the neoadjuvant setting or in metastatic patients. According to a recent meta‐analysis assessing ^18^F‐FES PET/CT safety and accuracy in patients with breast cancer recurrence or metastases, this technique is feasible, safe, and accurate, with a pooled sensitivity and specificity of 86% and 85%, respectively.^45^

### New Molecular Features Revealed by Targeted Tracers

In line with efforts to noninvasively decode the tumor phenotype, tracers other than ^18^F‐FDG are currently being investigated. None of these have been approved for use in clinical practice, as their usefulness and cost‐effectiveness are still under investigation in several clinical trials.^9^ Among these experimental tracers, the most promising for breast cancer assessment are 16α‐18F‐fluoroestradiol (^18^F‐FES), ^89^Zr‐trastuzumab, choline derivates, 3‐deoxy‐3‐[18F]fluorothymidine (^18^F‐FLT), and, recently, ^68^(Ga)‐FAP inhibitor (FAPI)‐46.^43^

#### ^89^ZR‐TRASTUZUMAB

How it works: The monoclonal antibody trastuzumab is labeled with ^89^Zr to identify breast cancer cells overexpressing the HER2 receptor, as is the case in luminal B (ER/PgR+, HER2+) and HER2+ (ER/PgR−, HER2+) breast cancer subtypes.^43^

**FIGURE 9:**
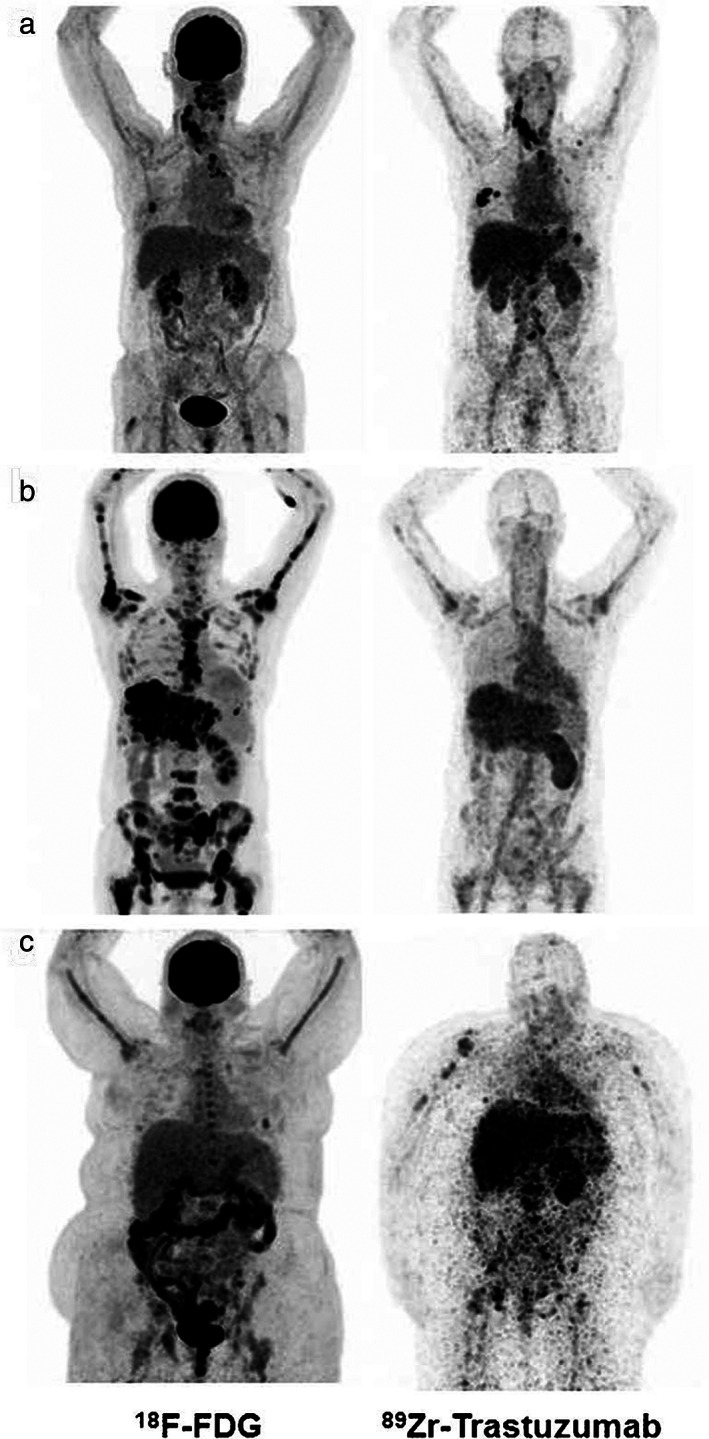
^18^F‐FDG (*left*) and ^89^Zr‐trastuzumab PET scans (*right*) of three patients: Example of a patient with a ^89^Zr‐trastuzumab PET scan considered HER2‐positive **(a)**, a ^89^Zr‐trastuzumab PET scan considered HER2‐negative **(b)**, and an ^89^Zr‐trastuzumab PET scan considered equivocal **(c)**. Reprinted under a Creative Commons (CC BY 4.0) license from: Bensch F, Brouwers AH, Lub‐de Hooge MN, de Jong JR, van der Vegt B, Sleijfer S, de Vries EGE, Schröder CP. ^89^Zr‐trastuzumab PET supports clinical decision making in breast cancer patients, when HER2 status cannot be determined by standard work up. Eur J Nucl Med Mol Imaging. 2018;45(13):2300–2306.

When it can be used: ^89^Zr‐trastuzumab can be used for identifying HER2+ breast cancer lesions as well as positive lymph nodes and metastasis. The possibility to noninvasively detect HER2 overexpression can have a huge impact in clinical practice, especially in patients with multifocal/multicentric tumors which can be difficult to characterize using core biopsy or when HER2 status cannot be determined with the standard workup as shown in Bensch et al's study.^46^ Figure 9 shows examples of ^18^F‐FDG and ^89^Zr‐trastuzumab PET scans in three patients whose HER2 status of remained unclear after the standard workup. In Bensch et al's study, ^89^Zr‐trastuzumab uptake was detected in HER2+ cases, whereas ^89^Zr‐trastuzumab uptake was not detected in HER2− cases. As molecular features of breast cancer can change during treatment because of tumor heterogeneity, ^89^Zr‐trastuzumab PET can be useful to assess the status of HER2 amplification in un‐responsive cases.

#### ^18^F‐FLT

How it works: Thymidine is a nucleotide that, differently from other nucleotides, can be incorporated only into DNA, which makes it a specific marker of cell proliferation via DNA synthesis. It was first labeled with ^11^C but, considering the short half‐life of ^11^C, ^18^F‐labeled thymidine has been considered as an alternative tracer. Its uptake in cancer cells occurs through either passive diffusion or equilibrative nucleoside transporters which are overexpressed in cells in response to 5‐fluorouracil.^43^

**FIGURE 10:**
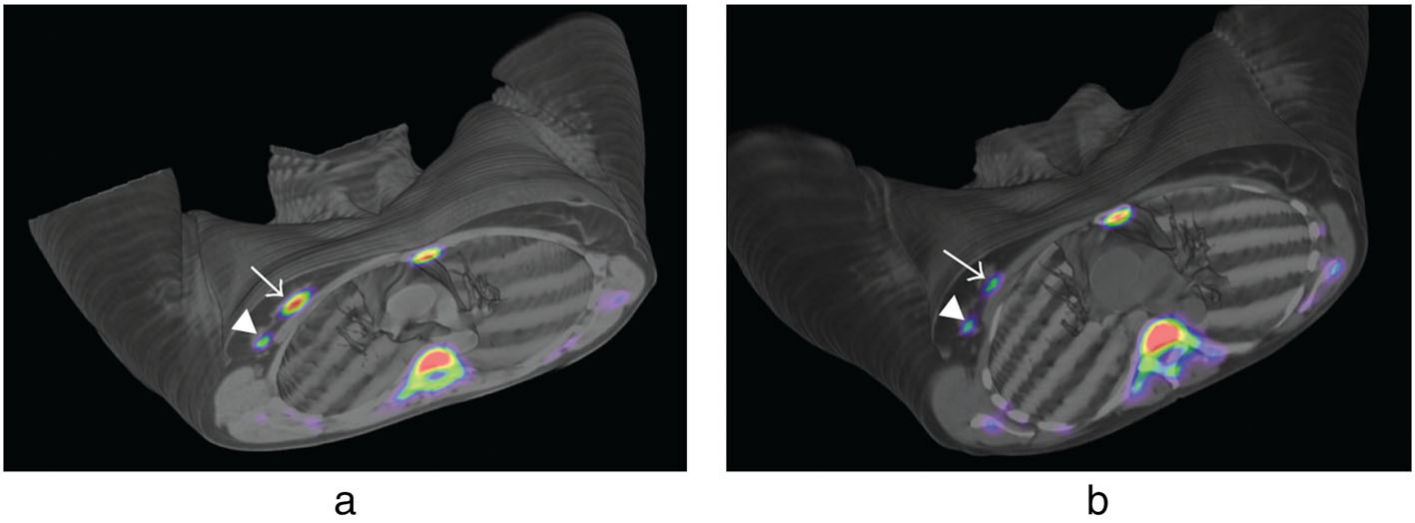
**(a)** Baseline PET/CT images obtained in a Biograph Duo LSO (Siemens) 75 minutes after injection of 405 MBq of ^18^FLT in a 47‐year‐old woman with a right‐sided infiltrating ductal carcinoma (SUVmax = 5.42) (arrow) and lymph node uptake (SUVmax = 1.85) (arrowhead). Physiological bone marrow uptake was identified. **(b)** PET/CT images obtained 75 minutes after injection of 529 MBq of ^18^FLT after one cycle of neoadjuvant therapy. SUVmax decreased to 3.57 in the primary tumor and to 0.80 in the lymph node, consistent with metabolic response. Reprinted under a Creative Commons (CC BY 4.0) license from: Peñuelas I, Domínguez‐Prado I, García‐Velloso MJ, Martí‐Climent JM, Rodríguez‐Fraile M, Caicedo C, Sánchez‐Martínez M, Richter JA. PET Tracers for Clinical Imaging of Breast Cancer. J Oncol. 2012;2012:710561.

When it can be used: ^18^F‐FLT has been found to highly correlate with the Ki67 proliferation index, with possible implications in the prediction of patient prognosis.^38^ Considering its biological underpinnings, pre‐ and intra‐treatment ^18^F‐FLT PET can be useful to predict response to cytotoxic chemotherapy, as has been supported in preliminary investigations^38^, ^47^ (Fig. 10).

#### ^68^GA‐FAPI‐46

How it works: Fibroblast activated proteins (FAP) are a sub‐group of activated fibroblasts which are not detectable in healthy tissues. It is hypothesized that these proteins may have a role in modulating the tumor microenvironment in terms of heterogeneity and plasticity, releasing factors responsible for the occurrence of cancer as well as for its invasion and biological behavior.^48^ On the other hand, some evidence also support a certain role of FAP in tumor suppression at early stages and have demonstrated their uptake in different types of cancer, including sarcoma, esophageal cancer, breast cancer, cholangiocarcinoma, and lung cancer.^49^

When it can be used: When labeled with ^66^Ga, FAPI can be used for breast cancer diagnosis and staging. FAPI labeled with ^66^Ga was recently introduced, and a preliminary investigation of ^66^Ga‐labeled FAPI in 19 breast cancer patients, of whom 18 had a primary tumor lesion and one had recurrent distant metastasis, was recently published.^50^ In this investigation, all breast cancers showed strong ^68^(Ga)‐FAPI‐46 uptake, with a mean maximum standard uptake value (SUVmax) of 13.9 (range, 7.9–29.9), similar to that of metastatic lymph nodes (mean SUVmax = 12.2; range, 3.3–22.4). These promising original findings encourage additional investigations to further define the clinical impact of this new tracer.

## Is There Value for Breast PET/MRI in Clinical Practice?

**FIGURE 11:**
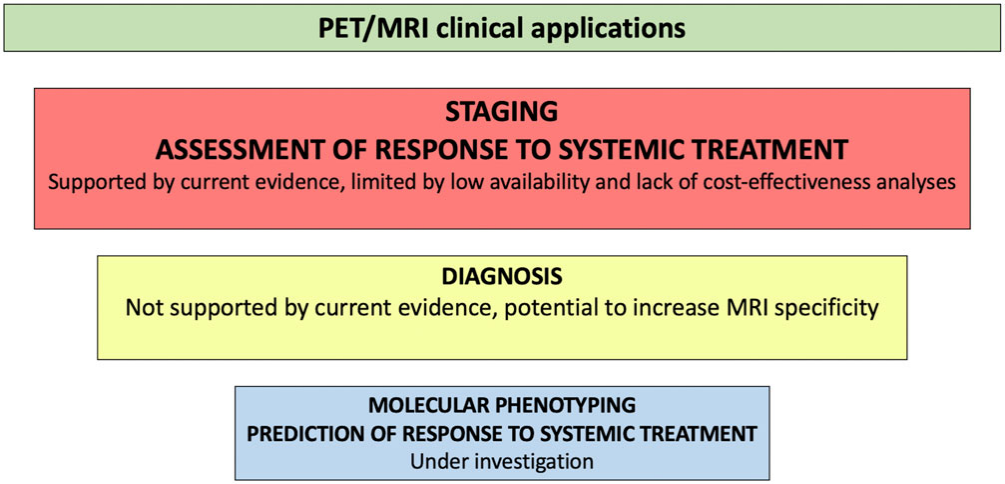
Clinical applications of hybrid PET/MRI in breast cancer in relation to their current evidence‐based status.

Since the first hybrid PET/MRI systems were installed, the first clinical investigations were mainly focused on comparing ^18^F‐FDG PET/MRI and PET/CT in terms of uptake and SUV estimation, and then on their diagnostic performance in the most relevant clinical scenarios. Overall, it can be said that PET/MRI is indisputably superior to PET/CT for the evaluation of the breast parenchyma. Regarding whole‐body staging, MRI can take advantage of the DWI technique for the detection of lymph node and bone metastasis, even if CT also provides useful information for bone lesions characterization (eg, assessment of cortical thickness and differentiation between sclerotic and lytic patterns). However, despite its advantages, the use of whole‐body MRI in clinical practice is still limited. Issues in which PET/CT is preferable are, of course, the evaluation of the lung parenchyma for the detection of small metastasis and the shorter acquisition time. In this section, clinical applications of breast PET/MRI will be illustrated, in order of clinical relevance based on the robustness of the available evidence. A summary of hybrid PET/MRI clinical applications is also provided in Fig. 11. To date, most investigations have been focused on the clinical applications of ^18^F‐FDG PET/MRI.

### Breast Cancer Staging

Considering the high resolution of MRI in breast tissue assessment, its leading role for pre‐operative breast cancer evaluation is undisputed, especially with the availability of specific MRI criteria for T staging.^51^ Nevertheless, at the molecular level, PET tracers like ^18^FDG and ^11^C‐choline recently showed promise to noninvasively stage breast cancer, demonstrating that PET can be used to monitor tumorigenesis from premalignancy to invasive carcinoma in mouse models.^52^

**FIGURE 12:**
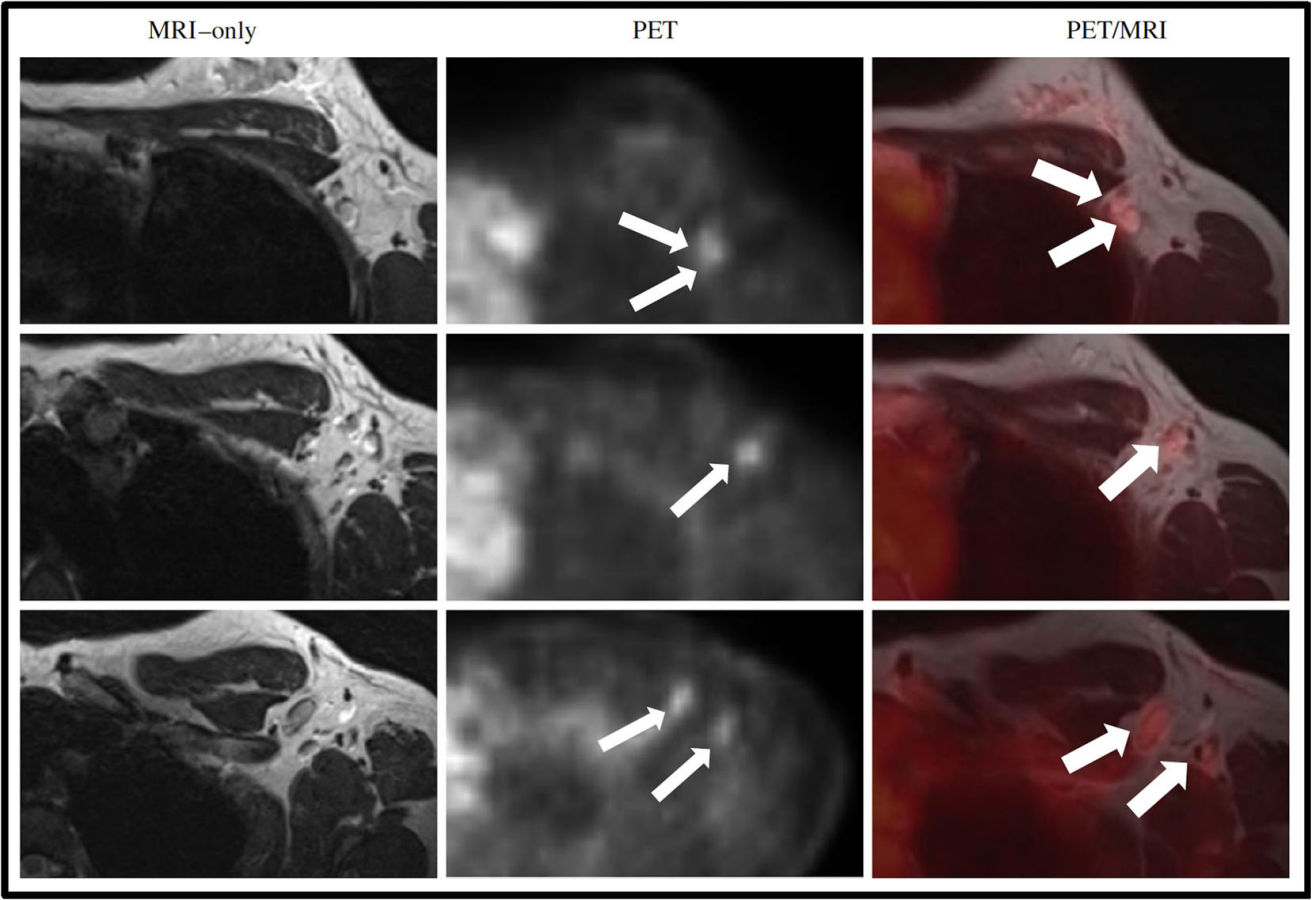
Images of a patient with no lymph nodes suspicious for metastases on MRI (T2w sequence is shown in the left column) and five axillary FDG hotspots suspicious for lymph node metastases on PET (small arrows, middle column). Adding PET information to MRI, resulted in five lymph nodes marked as suspicious for metastases (big arrows, right column). Reprinted under a Creative Commons (CC BY 4.0) license from: Goorts B, Vöö S, van Nijnatten TJA, Kooreman LFS, de Boer M, Keymeulen KBMI, Aarnoutse R, Wildberger JE, Mottaghy FM, Lobbes MBI, Smidt ML. Hybrid ^18^F‐FDG PET/MRI might improve locoregional staging of breast cancer patients prior to neoadjuvant chemotherapy. Eur J Nucl Med Mol Imaging. 2017;44(11):1796–1805.

Regarding N and M staging, many investigations have been conducted to define the usefulness of ^18^F‐FDG PET/MRI. Consequently, two systematic reviews and meta‐analyses were published in 2021 assessing the performance of ^18^F‐FDG PET/MRI for TNM staging, comparing it with ^18^F‐FDG PET and ^18^F‐FDG PET/CT as initial staging modalities, respectively.^53^, ^54^ As a result, PET/MRI showed excellent performance for the definition of the T, N, and M parameters, with an AUC of 0.96 (95% CI: 0.94–0.98), 0.96 (95% CI: 0.94–0.97), and 0.99 (95% CI: 0.98–1.00), respectively [53]. ^18^F‐FDG changed the tumor stage in 25% of cases (95% CI: 21%–30%) and, therefore, clinical management in 18% of cases (95% CI: 14%–23%) [54]. Percentages of variations were greater in more advanced stages like stage II (20%, 95% CI: 16%–24%) and III (34%, 95% CI: 27%–42%) compared to stage I (11%, 95% CI: 27%–42%). These data suggest that ^18^F‐FDG could be considered for routine breast cancer staging. Just as importantly, ^18^F‐FDG PET/MRI showed an added clinical value in 8 of 40 (20%) patients originally candidates for neoadjuvant chemotherapy, mainly due to the detection of bone and mediastinal lymph nodes metastases,^55^ changing the treatment plan in 10% of patients (Fig. 12).

In a more recent paper, a prospective comparison of nodal staging between CT, MRI, and ^18^F‐FDG PET/MRI was performed in 182 breast cancer patients. The authors found that ^18^F‐FDG PET/MRI outperformed CT and MRI in detecting axillary lymph node metastases at every level, revealing the presence of 193 lesions, whereas 123 and 104 lesions were detected by MRI and CT, respectively.^56^ Recent evidence supporting the good performance of PET/MRI for the preoperative assessment of axillary lymph nodes has led to dedicated prospective clinical trials aiming to compare PET/MRI with axillary surgery or sentinel lymph node biopsy in early and advanced breast cancer.^57^ This could allow for a further de‐escalation of surgical axillary approaches.

#### Take‐home Points

The combination of MRI and PET information is highly valuable for T, N and M staging in breast cancer patients, particularly in selecting patient candidates for neoadjuvant chemotherapy.

### Systemic Treatment Assessment and Prediction of Breast Primary

**FIGURE 13:**
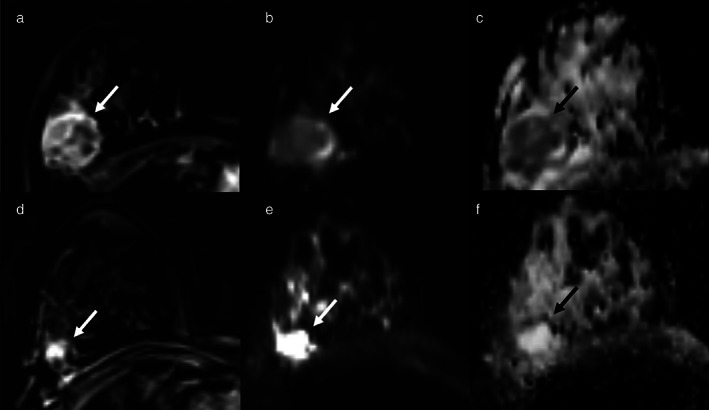
Example of early assessment of the response to neoadjuvant chemotherapy (NAC) using diffusion weighted imaging (DWI). examinations; (**a**–**c**) = pre‐NAC examinations; (**d**–**f**) = early assessment examination after two cycles of cytotoxic NAC. (**a** and **d**) = dynamic post‐contrast images; (**b** and **e**) = DWI images; (**c** and **f**) = ADC maps. A 37‐year‐old patient with a G3, triple negative invasive ductal carcinoma of the right breast (white and black arrows). Early assessment showed a reduction of tumor size along with increase of signal intensity on ADC maps **(c)** compared to the pre‐treatment examination **(f)**. Pathology after surgical resection revealed pathological complete response. Reprinted under a Creative Commons (CC BY 4.0) license from: Romeo V, Accardo G, Perillo T, Basso L, Garbino N, Nicolai E, Maurea S, Salvatore M. Assessment and Prediction of Response to Neoadjuvant Chemotherapy in Breast Cancer: A Comparison of Imaging Modalities and Future Perspectives. Cancers (Basel). 2021;13(14):3521.

**FIGURE 14:**
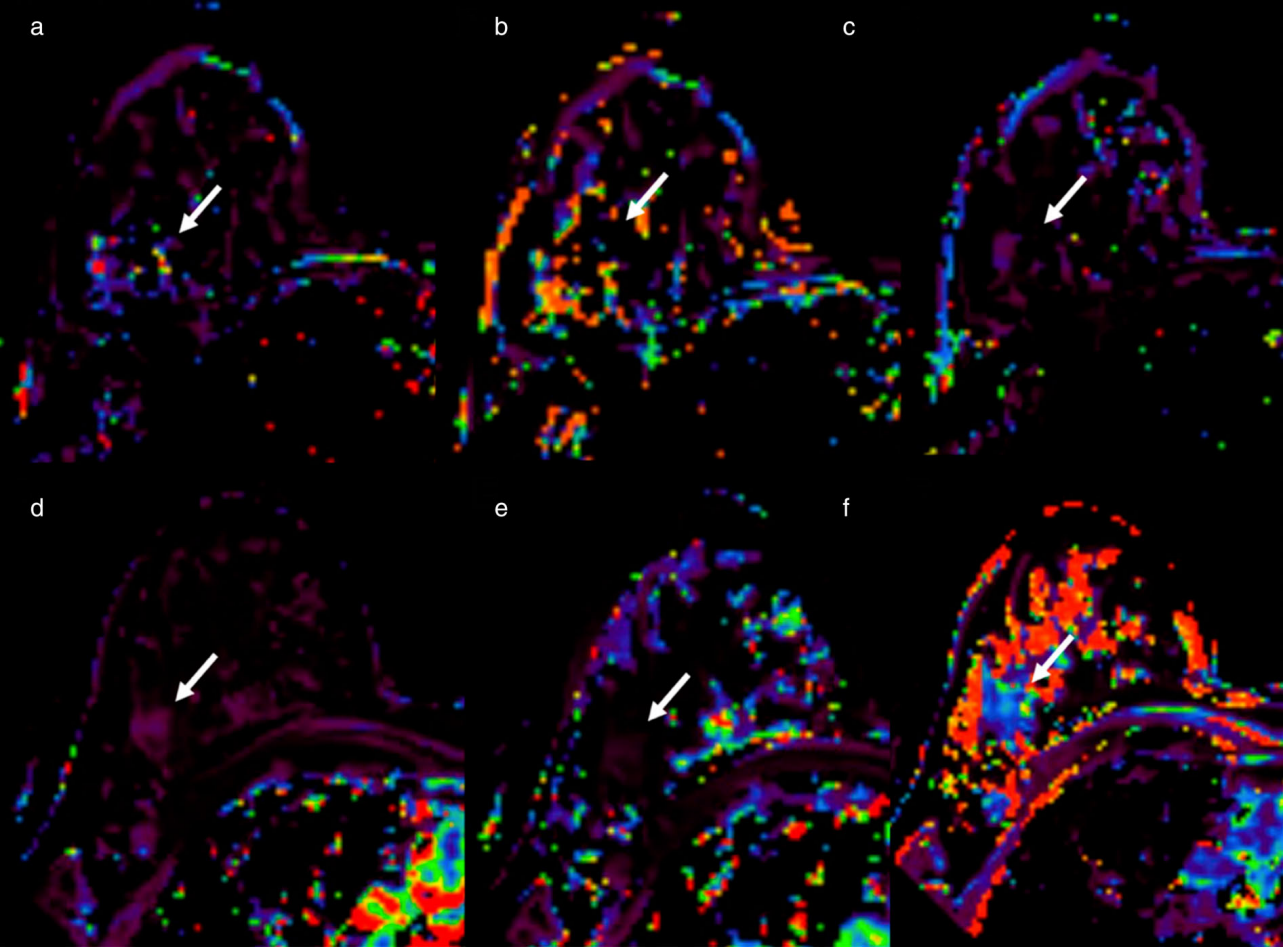
Example of early assessment of the response to NAC using dynamic contrast‐enhanced imaging (DCE‐MRI). 37‐year‐old patient with a G3, triple negative invasive ductal carcinoma of the right breast (arrows, same case shown in Figure 4). (**a**–**c**) = pre‐NAC examinations; (**d**–**f**) = early assessment examination after two cycles of cytotoxic NAC. Ktrans (**a** and **d**), Kep (**b** and **e**), and Ve (**c** and **f**) maps. Early assessment showed a reduction of Ktrans (286 vs. 83.9 min^−1^) and kep (91.49 vs. 20.14 min^−1^ × 100) with a slight increase of Ve (275.34 vs. 308.08 × 1000) signal intensity on ADC maps **(c)** compared to the pre‐treatment examination **(f)**. Pathological complete response was proved at pathology examination after surgical resection. Reprinted under a Creative Commons (CC BY 4.0) license from: Romeo V, Accardo G, Perillo T, Basso L, Garbino N, Nicolai E, Maurea S, Salvatore M. Assessment and Prediction of Response to Neoadjuvant Chemotherapy in Breast Cancer: A Comparison of Imaging Modalities and Future Perspectives. Cancers (Basel). 2021;13(14):3521.

**FIGURE 15:**
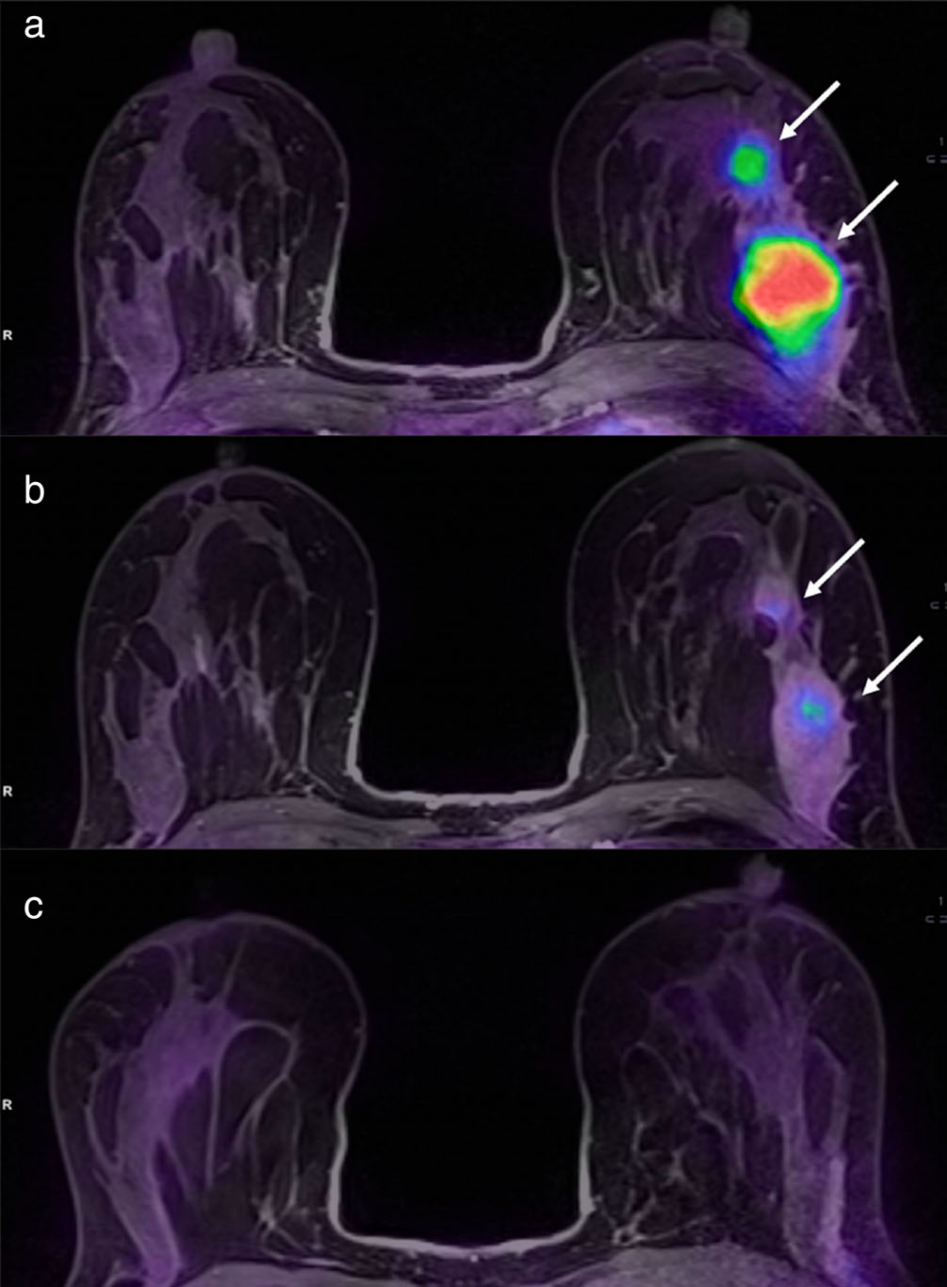
A 36‐year‐old patient with left breast cancer undergoing NAC. Fused PET/MRI images acquired before **(a)**, during **(b)**, and after **(c)** NAC are shown. While a slight reduction of the tumor and its satellite nodule (white arrows in **b**) is appreciable, ^18^FFDG uptake is significantly reduced after the second cycle of chemotherapy **(b)** as compared to the pre‐treatment evaluation **(a)**. The tumor was not detectable at the post‐treatment evaluation **(c)**. Pathology after surgery demonstrated a complete response. Reprinted under a Creative Commons (CC BY 4.0) license from: Romeo V, Accardo G, Perillo T, Basso L, Garbino N, Nicolai E, Maurea S, Salvatore M. Assessment and Prediction of Response to Neoadjuvant Chemotherapy in Breast Cancer: A Comparison of Imaging Modalities and Future Perspectives. Cancers (Basel). 2021;13(14):3521.

The assessment but particularly the prediction of response to systemic treatment can take advantage of the ability of ^18^F‐FDG PET/MRI to provide morpho‐functional evaluation; this currently represents the most challenging and ambitious task of ^18^F‐FDG PET/MRI research.^19^ Although response assessment criteria based on changes in tumor size (eg, Response Criteria in Solid Tumors, RECIST) have been employed both in the clinical routine and in clinical trials, new functional criteria are needed to cater to the demands of new biological and targeted treatments. What is more, functional assessment has the potential to noninvasively detect biological tumor changes before morphological changes can be appreciated, for example, in terms of cellularity (DWI/ADC), neoangiogenesis, and glucose uptake in response to a systemic treatment (Figs. 13, 14, 15).

In light of this, PET Response Criteria in Solid Tumors (PERCIST) have been proposed for a standardized assessment of FDG Uptake, in which SUV is normalized by lean body mass and abbreviated as SUL.^58^ According to such criteria, an uptake decrease by 30% is considered an index of tumor response, with a minimum SUL absolute difference of 0.8.^59^ Recent evidence also suggests the feasibility of PERCIST for treatment monitoring of metastatic breast cancer with a possible clinical decision‐making role as to whether or not to stop unresponsive chemotherapy schedules early.^60^ Functional assessment is also promising to predict response to treatment at baseline evaluation, thereby aiding in the selection of patients for neoadjuvant chemotherapy; for those patients predicted to have a low probability of achieving pathological complete response following neoadjuvant chemotherapy, they can be selected for surgical excision instead, avoiding unnecessary toxicity and psychologic distress.

While the role of MRI and ^18^F‐FDG PET/CT in assessing response to treatment has been widely explored and consolidated over the last years, with both having comparable high accuracy values, few investigations have assessed the specific role of hybrid ^18^F‐FDG PET/MRI. In a recent retrospective analysis including 74 patients, ^18^F‐FDG PET/MRI showed a sensitivity and specificity of 72.2% and 78.6%, respectively, in diagnosing complete response to neoadjuvant chemotherapy, with both sensitivity and specificity reaching 100% in both hormone‐ positive and ‐negative patients.^61^

Other published studies in the past few years have explored the role of hybrid ^18^F‐FDG PET/MRI particularly in the early prediction of the response to neoadjuvant chemotherapy, as the possibility to simultaneously collect and combine collected functional MRI and PET data make it possible to employ such data in the detection of biological signs of response at their early onset. In a preliminary paper, tumor size, diffusion (ADC_mean_), perfusion (K^trans^, K_ep_, v_e_, iAUC), and metabolic (SUV_max_, metabolic tumor volume) data were collected from patients undergoing cytotoxic or hormone neoadjuvant chemotherapy.^7^ In patients classified as having partial response, a decrease in functional parameters was observed, which was more pronounced after cytotoxic neoadjuvant chemotherapy rather than hormone neoadjuvant chemotherapy. In another study, MRI parameters (peak enhancement ratio, ADC_min_, choline signal‐to‐noise ratio) combined with PET parameters (SUV_max_, total lesion glycolysis) were acquired at baseline and during treatment (after the first or second neoadjuvant chemotherapy cycle). As a result, hybrid markers such as Δ% SUV_max_/Δ% ADC_min_, and Δ% total lesion glycolysis/Δ% ADC_min_ showed a high accuracy in predicting the final response to neoadjuvant chemotherapy (AUC of 0.976 and 0.905, respectively).^62^ Similarly, the combination of total lesion glycolysis from ^18^F‐FDG PET and signal enhancement ratio from MRI was shown to be predictive of response to neoadjuvant chemotherapy after the first cycle in 26 breast cancer patients, achieving a sensitivity of 100% and a specificity of 71.4%.^63^

#### Take‐home Points

Preliminary investigations suggest a possible and ambitious role of ^18^F‐FDG PET/MRI for the early prediction of response to neoadjuvant chemotherapy. However, investigations are currently limited to small cohorts of patients and standardized methods for MRI parameters calculation have yet to be defined.

### Molecular Phenotyping

Breast cancer may present with different molecular subtypes in relation to the expression of hormone receptors and HER2 amplification/overexpression. The definition of these molecular patterns is essential for establishing the right treatment, i.e., upfront surgery or different neoadjuvant chemotherapy approaches.

Currently, molecular characterization is performed by analyzing a sample obtained from core biopsy. However, this means that only a limited tumor sample is obtained and analyzed. Thus, molecular biomarkers revealed by core biopsy may not be the same as that for the entire lesion, with important implications on patient management and prognosis.^64^ Moreover, molecular patterns may change during chemotherapy and affect tumor resistance. Thus, molecular characterization may be required even during treatment, to establish whether the chemotherapy schedule should be changed to a more effective one. With these considerations, efforts have been made to find correlations between imaging data and tumor molecular features. In regards to ^18^F‐FDG PET/MRI‐derived imaging data, SUV_max_ and ADC_mean_ have been shown to correlate with tumor aggressiveness in terms of Ki‐67 expression, tumor grade and histological subtypes (*P* < 0.001).^65^

Recently, more sophisticated analyses have been conducted to determine correlations between ^18^F‐FDG PET/MRI‐derived imaging data and circulating biomarkers such as miRNA, which are released into the bloodstream by cancer cells. This might be helpful to noninvasively identify patients with breast cancer. Incoronato et al, found correlations between ADC_mean_, K_epmean_, and SUV_max_ with circulating miRNA “MiR‐143‐3p” in their study in 77 treatment‐naïve breast cancer patients.^66^ A further study by the same research group in 50 breast cancer patients found that ADC_mean_, metabolic parameters (SUV; and the peak lean body mass corrected, SUV_max_, SUL), and perfusion parameters (K^trans^, K_ep_) discriminated luminal A subtypes from luminal B and non‐luminal subtypes, with K^trans^ and SUV_max_ being the best parameters for predicting patient prognosis.^67^

Similarly, in another study in 21 breast cancer patients, perfusion (K_ep_) and metabolic (SUV_max_) parameters were found to be higher in hormone‐positive tumors compared to hormone‐negative tumors, while HER2+ lesions showed higher ADC_mean_, K_ep_, and SUV_max_ values than HER2− lesions.^68^

#### Take‐home Points

Initial evidence supports the possibility of hybrid PET/MRI to noninvasively predict molecular features of breast cancer, which is an extremely attractive prospect. However, investigations which have been conducted toward this goal are still exploratory, and more robust evidence are needed. To achieve this goal, imaging biobanks consisting of both DICOM images of cancer patients and corresponding biological data are being built.^69^ This will allow the collection of a large amount of shared data and enable the achievement of more robust results.

### Diagnosis

Due to the overall high sensitivity of ultrasound, digital mammography/tomosynthesis and MRI, ranging from 93.3% to 98.2%,^70^ ^18^F‐FDG PET/MRI is currently not recommended for diagnosing breast cancer. In addition to radiation exposure, ^18^F‐FDG PET/MRI has a low sensitivity in small lesions and have resulted in both false‐negative and false‐positive findings as benign lesions can show tracer uptake. Currently, no established SUV thresholds exist to make breast lesion uptake assessment more objective. However, the addition of PET has been shown to increase the specificity of MRI, from 53% to 97% in Moy et al^71^ and from 67% vs. 100% in Botsikas et al,^72^ and to improve diagnostic performance (from 86% to 93.5%) when used within a multiparametric approach combining DCE‐MRI, DWI, MRS, and PET.^73^ Thus, the development of advanced strategies allowing for simultaneous characterization, molecular profiling and staging for breast cancer diagnosis would be useful for patient management. A multiparametric, noninvasive PET/MRI strategy for breast cancer diagnosis would also be appealing for the characterization of incidental and additional breast lesions, especially in clinically suspected multi/focal or multicentric tumors.

#### Take‐home Points

While ^18^F‐FDG PET/MRI is currently not indicated for breast cancer diagnosis, its use could improve the diagnostic accuracy of MRI and, in the future, allow for less invasive comprehensive diagnostic and staging strategies.

## What's Next?

A new possibility for cancer imaging research came with the rise of informatics applications involving the evaluation and quantification of pixel distribution at different complexity levels (eg, characteristics of single pixels, relationship between pairs of pixels and the relationship between neighboring pixels). Artificial intelligence (AI) algorithms have been used to extracting quantitative data depicting image heterogeneity not accessible by human readers and then using such quantitative data, called “radiomics features,” to build predictive models.^74^ AI algorithms, mainly machine and deep learning algorithms, are trained and tested on varied datasets to make predictions; of note, their diagnostic ability improves with experience. As there are many currently unpredictable applications of AI, including if and how AI algorithms can co‐exist with human radiologists, AI has rapidly become a hot topic in the field of oncologic imaging in the recent years. As far as the evidence goes, radiomics and AI applications are powerful and high‐performing in different predictions tasks, and are potentially able to further empower the detection of molecular and prognostic data provided by functional imaging.^75^, ^76^

Hybrid PET/MRI is one of the most promising and attractive imaging modalities for radiomics and AI applications. While many studies have applied radiomics and AI to PET and MRI for breast cancer assessment with interesting and promising findings for many outcomes,^77^, ^78^ to date, there are only a few studies which have applied radiomics and AI to hybrid PET/MRI for breast cancer assessment. The first paper to report the results of hybrid PET/MRI radiomics for breast cancer assessment explored the contribution of different combinations of radiomic features and quantitative diffusion, perfusion, and PET parameters for discriminating 19 benign from 101 malignant breast lesions. A support vector machine with 5‐fold cross validation yielded the highest accuracy (AUC = 0.983) when both quantitative parameters (MTT and ADC) and radiomic features extracted from PET and ADC images were selected, outperforming an expert breast radiologist and a nuclear medicine physician (AUC = 0.868), albeit the difference was not statistically significant (*P* = 0.508).^79^ In another study, a support vector machine with 5‐fold cross validation was also employed for the prediction of breast cancer subtypes, tumor grade, nodal status, and presence of distant metastasis in 124 breast cancer patients.^80^ The best results for the prediction of hormone receptor, nodal status, and proliferation rate were found based on all MRI and PET data, with an AUC of 0.87 for estrogen receptor status, 0.88 for progesterone receptor status, and 0.997 for Ki‐67, respectively. PET features yielded the best performance for the assessment of tumor grade (AUC = 0.71), while the combination of MRI and PET features yielded the best performance for the prediction of lymph node status (AUC = 0.81) and the presence of distant metastases (AUC = 0.99). Similarly, in another study, ^18^F‐FDG PET/MRI‐derived features demonstrated a good performance for the prediction of pathological complete response after neoadjuvant chemotherapy (AUC = 0.80, 0.89, and 0.94 for the entire cohort, hormone+/HER2− patients, and triple negative/HER2+ patients, respectively).^81^ Of note, while findings have been very encouraging, current studies are limited by the lack of external validation, which is a hard task considering the low availability of hybrid PET/MRI scanners and therefore the limited number of potential patients that can be included in the studies.

### Take‐Home Points

Radiomics and AI applications represent further promising efforts to extract as much information as possible from tumors, trying to decode the tumor phenotype and predict the tumor's biological behavior. Their applications to hybrid PET/MRI are still in their infancy, requiring the standardization of AI methods as well as the availability of larger patient samples to externally validate the developed models and assess their generalizability.

## Hybrid PET/MRI in the Real World: Strengths and Weaknesses

While the current evidence is encouraging on the usefulness and potential of hybrid PET/MRI for the assessment of breast cancer, some practical aspects have to be acknowledged. The widespread use of this advanced technology is currently jeopardized by its high procurement and maintenance costs. While combining PET with MRI instead of CT allows for a significant reduction of radiation exposure, which would be highly beneficial for younger or radiation‐susceptible patients including carriers of germline mutations in DNA‐damage repair pathway genes (BRCA1, BRCA2, CHEK2, and ATM),^82^, ^83^ MRI involves long acquisition times. However, several efforts are currently ongoing to shorten both breast PET/MRI and, particularly, whole‐body PET/MRI acquisition protocols.

## Closing Remarks

Although the wide use and successful implementation of hybrid PET/MRI are currently jeopardized by its high costs and limited availability, it is the most promising imaging modality for breast cancer assessment, providing a fully integrated morphologic and functional imaging assessment. Indeed, the simultaneous integration of MRI with PET expands the applications of both modalities, even as new, highly specific PET tracers are being developed for breast cancer assessment. Research investigations on hybrid PET/MRI, including in multicenter settings and in the context of clinical trials, are currently ongoing, and more such investigations are strongly encouraged in order to define the clinical role of this innovative and compelling imaging modality.

## Conflicts of Interest

Katja Pinker received payment for activities not related to the present article including lectures and service on speakers bureaus and for travel/accommodations/meeting expenses unrelated to activities listed from the European Society of Breast Imaging (MRI educational course, annual scientific meeting), the IDKD 2019 (educational course), and Siemens Healthineers. At the time of the writing of this article, Katja Pinker was also a consultant for Genentech Inc., Merantix Healthcare, and AURA Health Technologies GmbH. Thomas H. Helbich received payment for activities not related to the present article including lectures and service on speakers bureaus and for travel/accommodations/meeting expenses unrelated to activities listed from the European Society of Breast Imaging, the IDKD 2019 (educational course), and Siemens Healthineers, Guerbet, Novomed.
